# Trends in Antidepressant, Anxiolytic, and Cannabinoid Use Among Italian Elite Athletes (2011–2023): A Longitudinal Anti-Doping Analysis

**DOI:** 10.3390/sports13070233

**Published:** 2025-07-16

**Authors:** Mario Ruggiero, Leopoldo Ferrante, Domenico Tafuri, Rosaria Meccariello, Filomena Mazzeo

**Affiliations:** 1 Department of Medical, Human Movement and Well-Being Sciences, University of Naples Parthenope, 80133 Naples, Italy; mario.ruggiero005@studenti.uniparthenope.it (M.R.); domenico.tafuri@uniparthenope.it (D.T.); rosaria.meccariello@uniparthenope.it (R.M.); 2 Department of Economics, Law, Cybersecurity and Sports Sciences, University of Naples Parthenope, 80035 Nola, Italy; leopoldo.ferrante@collaboratore.uniparthenope.it

**Keywords:** elite athletes, sport, mental health, antidepressant, anxiolytics, cannabinoids, delta-9-tetrahydrocannabinol (THC), cannabidiol (CBD), antidoping, Coronavirus Disease 19 (COVID-19) impact, drugs

## Abstract

Mental health disorders, particularly depression and anxiety, have become increasingly prevalent among elite athletes, exacerbated by factors such as competitive pressure and the Coronavirus Disease 19 (COVID-19) pandemic. This study analyzes trends in the use of antidepressants, anxiolytics, and cannabinoids (delta-9-tetrahydrocannabinol (THC)/cannabidiol (CBD)) among Italian athletes from 2011 to the first half of 2023 (FH2023), referring to anti-doping reports published by the Italian Ministry of Health. Data from 13,079 athletes were examined, with a focus on non-prohibited medications, banned substances, and regulatory impacts, including threshold adjustments for THC since 2013 and the legalization of CBD. The results show fluctuating use of antidepressants/anxiolytics, with peaks in 2021 and the FH2023, coinciding with post-pandemic awareness. Positive THC cases rose following regulatory changes, reflecting socio-cultural trends. Gender disparities emerged, with THC use predominantly among males (e.g., nine males vs. one female in 2013), though female athletes were underrepresented in testing. This study highlights the need for personalized, evidence-based strategies that balance therapeutic efficacy and anti-doping compliance. Clinicians should carefully consider prescribing selective serotonin reuptake inhibitors (SSRIs) and benzodiazepines to address depression and anxiety and should monitor the risks of CBD contamination. Future research should adopt longitudinal, gender-sensitive approaches to refining guidelines and combating stigma in professional sports.

## 1. Introduction

Mental disorders represent the most impactful pathologies worldwide on health care systems. In 2019, depression and anxiety ranked among the top 25 disabling conditions and were the most prevalent mental disorders [1,2]. Coronavirus Disease 19 (COVID-19), with its consequences such as lockdowns, stay-at-home orders, school and business closures, and reduced social interactions, negatively affected these numbers, increasing the prevalence of major depressive disorder and anxiety disorders in 2020. Santomauro et al. reported, 3152.9 cases of major depressive disorder and 4802.4 cases of anxiety disorders per 100,000 inhabitants, globally [3]. As before the pandemic, women were more affected than men, showing an even greater prevalence difference [3]. Additionally, young people were more affected by mental disorders than older adults [4]. In the athletic context, stressors such as event cancellations, isolation, and the disruption of training routines may have contributed to increased psychotropic medication use in the years following the pandemic.

**Figure 1:**
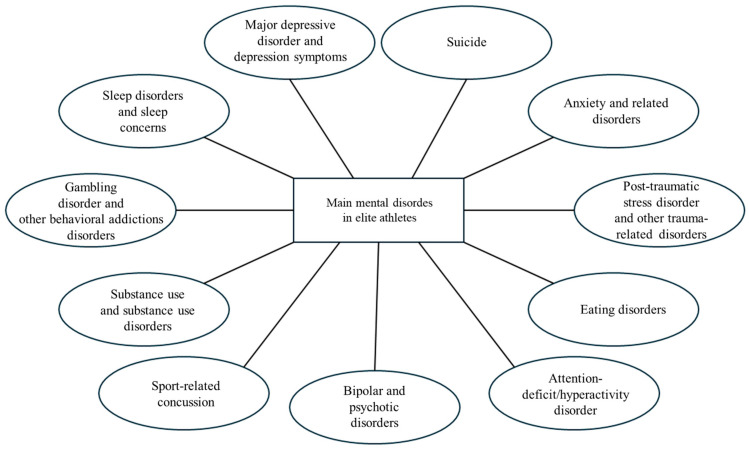
Major mental disorders that afflict elite athletes.

The connection between physical and mental well-being has been rooted in Western culture since antiquity, as recalled by Juvenal in his famous aphorism “*Mens sana in corpore sano*” (“A sound mind in a sound body”). Several studies confirm this intuition, demonstrating how physical activity reduces the risk of developing mental disorders [5,6,7,8]. However, in elite athletes, factors such as competitive pressure, psychophysical overload, and injuries reverse this correlation [9,10]. The main mental disorders that afflict elite athletes are summarized in Figure 1 [10].

Athletes are more affected by mental disorders than the general population, with higher prevalence of depression and anxiety, especially in high-pressure competitive sports [10]. In addition, these disorders are underreported [11,12]. Barriers to diagnosis and treatment include clinicians’ hesitancy to label athletes with mental illnesses, athletes’ avoidance of help-seeking behaviors, and persistent stigma framing mental disorders as personal weakness [9]. Research indicates bidirectional links between mental health and athletic performance [13]. For instance, 68% of Canadian swimmers qualifying for the 2012 Olympics reported major depressive episodes in the three years preceding the trials, correlating with poorer competition metrics (time, scores, and rankings) [14]. Post-selection data from the 2016 Olympics revealed that 80% of 50 athletes experienced depressive episodes within 12 months of qualification [15]. Drew et al. further demonstrated that mental health disorders during Olympic participation predicted performance declines and somatic symptoms [16]. The latest available data, also cited in the latest 2024 Paris Olympics, report that 33.6 percent of elite athletes suffer from depression or anxiety [17,18]. Injury patterns also reflect mental health status: anxiety correlates with injury frequency, while tension and negative affect predict injury severity [19]. Johnson et al. identified stress, anxiety, distrust, and poor coping strategies as key predictors of injuries, with a model accuracy of 67% [20].

The management of mental disorders in elite athletes requires a holistic and personalized approach, centered on the athlete, but also capable of integrating all dimensions of well-being (i.e., emotional, cognitive, physical, social, spiritual, and environmental well-being). It is essential to develop targeted therapeutic strategies that (a) adopt evidence-based multidisciplinary interventions, (b) consider the athlete’s specific context (sports, cultural, and geographical context), and (c) balance symptom management with the promotion of overall well-being. This model should be based on the best available scientific evidence and adapt to the peculiarities of the professional sport practiced [10].

Psychotherapy is effective for depression and anxiety in athletes but remains underused [21]. Cognitive behavioral therapy is the gold standard for the general population in addressing these mental disorders, but there are still insufficient athlete-specific studies [22]. Elite athletes present unique challenges for psychotherapists. The difficulty in distinguishing between overtraining symptoms and depression [23], combined with personality traits such as marked competitiveness and entitlement, requires specialized approaches, either psychotherapeutic or pharmacological [24].

Although psychotherapy may be sufficient for mild to moderate mental health symptoms [25], pharmacological therapy becomes necessary for more severe cases [26]. However, the use of medications in athletes is subject to the anti-doping regulations of the World Anti-Doping Agency (WADA) [27,28,29,30]. The World Anti-Doping Code (WADC) [31] contains the list of prohibited substances and methods, known as the Prohibited List [32]. Athletes may request a Therapeutic Use Exemption (TUE) if they can demonstrate that all the following conditions are met: (a) established medical need; (b) no valid therapeutic alternatives; (c) no performance advantage; (d) minimum effective dose [33]. The main medications prescribed to treat anxiety and depression are not included in the Prohibited List [32], as there is insufficient evidence of ergogenic doping effects. However, when prescribing these medications to athletes, it must be considered that these substances may affect performance and cause side effects [34]. Indeed, some athletes use cannabis to enhance performance or for recreational purposes [35]. While the WADA prohibits cannabis (specifically its main psyco-active cannabinoid, delta-9-tetrahydrocannabinol (THC)) in all competitive sports during events as of 2004 [36], cannabidiol (CBD)-based products—when containing ≤0.1% THC—have been permitted since 2018 [37]. Since 2013, the WADA has raised the threshold for THC to 150 ng/mL [38], limiting positive results to in-competition use: in fact, the new value excludes false positives resulting from post-administration drug residues [39]. CBD demonstrates anxiolytic, anti-inflammatory, and neuroprotective properties with minimal side effects [40,41]. Conversely, THC remains a banned substance due to its potential to impair reaction time and motor coordination [36].

In light of these considerations, this study aims to assess trends (increase, decrease, or stability) in the use of classical antidepressants and anxiolytics and CBD among Italian athletes, utilizing anti-doping reports published by the Italian Ministry of Health from 2011 to the first half of 2023 (FH2023) [42]. Given the complexity of treating mental health in athletes, this analysis will focus on pharmacological strategies for depression and anxiety, including gender-based consumption patterns, focusing on efficacy, risks, and anti-doping regulations.

## 2. Materials and Methods

In the present study, data were extracted from the “Reporting System Doping Antidoping Archives,” considering 13 annual reports (2011–FH2023) published by the Italian Ministry of Health [42]. The tests involved events organized both by Federazioni Sportive Nazionali (FSN), Discipline Sportive Associate (DSA), and Enti di Promozione Sportiva (EPS). These reports examined 13,079 professional athletes who underwent official anti-doping testing in Italy (average: 1006.08/year). The analyzed sections were those dedicated to the use of non-prohibited medications and/or health products, with particular attention to the reported use of antidepressants and anxiolytics, as well as the use of substances included in the WADA Prohibited List [32]—especially focusing on the number of athletes who tested positive for THC or its metabolites. The 2010 report was not included in this study because it did not provide analyses on the use of non-doping medications and/or health products.

It should be noted that samples received by analytical laboratories from 11 May 2013 onward were subject to the new threshold level for THC, which increased from 15 ng/mL (Decision Limit = 19 ng/mL) to 150 ng/mL (Decision Limit = 175 ng/mL). This decision by the WADA was made to avoid false positives resulting from post-administration drug residues [38].

The number of athletes who used THC was expressed as a percentage relative to the total sample examined, the number of athletes who tested positive in anti-doping tests, and the number of prohibited substances used.

This observational study analyzed longitudinal administrative records without intervention. While enabling population-level trend assessment, this design inherits limitations of secondary data including variable completeness and contextual constraints.

## 3. Results

The results of the analysis of anti-doping reports from 2011 to the FH2023 are presented below. The data include the number of athletes undergoing anti-doping testing; the use of non-prohibited medications, specifically antidepressants and anxiolytics; and positive test results for THC, stratified by sex. Trends over time were examined to assess changes in the use of these substances among Italian athletes.

Table 1 shows the data from 2011 to the FH2023, from the “anti-doping reporting systems” [42], displaying the number of athletes who underwent anti-doping tests in Italy and the number of athletes who reported using anxiolytics or antidepressants.

**Figure 2:**
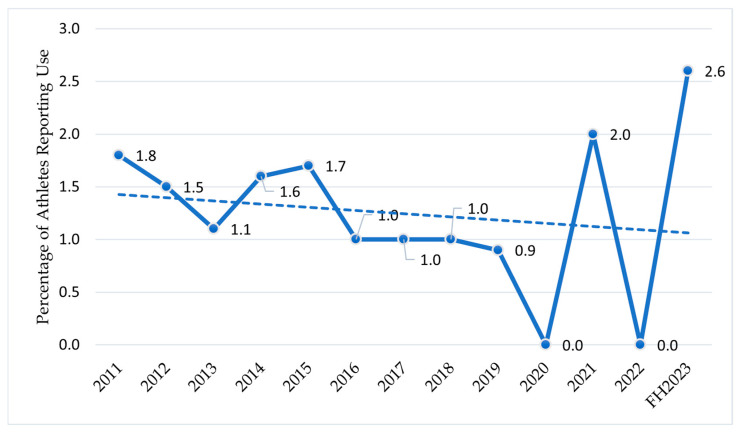
The use of anxiolytics or antidepressants in athletes from 2011 to the FH2023 in Italy. Athletes who reported using anxiolytics or antidepressants. Data are expressed as the % ratio between the number of athletes who reported using anxiolytics or antidepressants and the total number of athletes tested for doping in the selected year/period.

Figure 2 shows the percentages of athletes who reported using anxiolytics or antidepressants relative to the number of athletes who underwent anti-doping analyses from 2011 to the FH2023 with the trendline.

Table 2 shows the number of athletes who tested positive and the number of prohibited substances detected during anti-doping tests from 2011 to the FH2023. Among the prohibited substances detected by anti-doping, data on THC-positive athletes are reported in Table 2 and further stratified by sex. However, for the years 2011 and 2012, the gender division is not shown in the reports.

**Figure 3:**
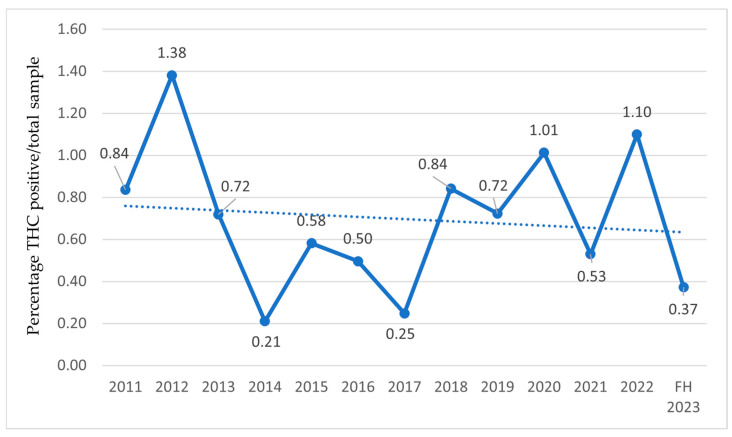
THC-positive athletes relative to the total number of examined samples from 2011 to the first half of 2023 (FH2023) in Italy. Data are expressed as the percentage ratio between the number of THC-positive athletes and the total number of examined samples.

**Figure 4:**
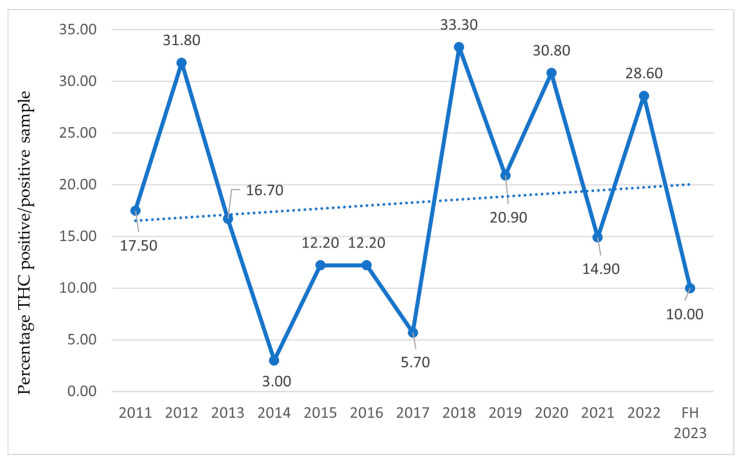
THC-positive athletes relative to the total number of positive antidoping tests from 2011 to the first half of 2023 (FH2023) in Italy. Data are expressed as the percentage ratio between the number of THC-positive athletes and the total number of tested positive on antidoping tests.

**Figure 5:**
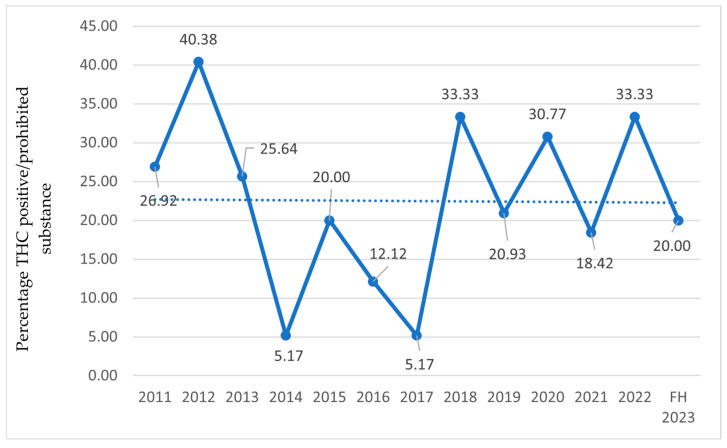
THC-positive athletes relative to the total number of prohibited substances from 2011 to the first half of 2023 (FH2023) in Italy. Data are expressed as the percentage ratio between the number of THC-positive athletes and the number of prohibited substances.

In the following charts (Figure 3, Figure 4 and Figure 5), the percentage of athletes with detected THC use is reported relative to the total examined sample (Figure 3), athletes who tested positive on antidoping tests (Figure 4), and the number of prohibited substances with trendlines (Figure 5).

## 4. Discussion

This study provides a longitudinal analysis of pharmacological trends for depression and anxiety among Italian elite athletes from 2011 to the FH2023. Using anti-doping reports from the Italian Ministry of Health, we evaluated the prevalence of antidepressants, anxiolytics, and THC/CBD use in a high-performance population, where mental health management intersects with stringent anti-doping regulations and unique physiological demands [27,28,29,30].

The COVID-19 pandemic exacerbated mental health challenges globally, particularly in competitive environments, yet its impact on pharmacological choices in sports remains underexplored [3]. Our data reveal fluctuating patterns in antidepressant and anxiolytic use, accompanied by an increase in THC-positive tests, likely reflecting broader socio-cultural shifts in substance use and stress management strategies [35,36]. These trends highlight the tension among clinical needs, performance optimization, and regulatory compliance [10,26].

Our findings align with emerging evidence of disproportionate barriers to mental health care access in athletes, including stigma and diagnostic complexities (e.g., overlapping symptoms of overtraining and depression) [9,23]. The underreporting of mental disorders, combined with the risks of ergolytic effects from psychotropic medications, underscores the need for personalized therapeutic frameworks [34]. In the following sections, we analyze these critical issues through the lens of pharmacotherapy for depression and anxiety, contextualizing the results in Italian/international contexts, anti-doping policies, and athlete-specific risks [10,31,43,44].

### 4.1. Pharmacotherapy of Depression in Athletes

The treatment of depression in athletes must carefully evaluate clinical benefits and risks to health and performance, balancing symptomatic efficacy with physical and regulatory implications [26,34].

Studies analyzing the general Italian population show an increasing trend in the consumption of antidepressants over the period of 2008–2022. Oscoz-Irurozqui et al. [45] indicated a significant Average Annual Percent Change (AAPC) of +2.31% in overall antidepressant consumption.

Specifically, selective serotonin reuptake inhibitors (SSRIs) and serotonin–norepinephrine reuptake inhibitors (SNRIs) showed significant increases, while tricyclic antidepressants (TCAs) decreased. It is reasonable to hypothesize that similar trends observed in the general population might also be reflected within the athlete population. SSRIs are a widely used class of antidepressants, also prescribed to athletes [26,34]. However, Roelands et al. (2008) [46] found negative effects on performance, despite this drug class being associated with energizing effects. Fluoxetine remains the drug of choice for elite athletes, as it is the active ingredient that has demonstrated the least deterioration in performance [47].

TCAs and mirtazapine commonly cause sedation and weight gain as side effects [48]. Prescriptions of tricyclic antidepressants should be limited for athletes because they may cause supraventricular and ventricular arrhythmias [49] and are not recommended for patients who sweat excessively, such as athletes [50].

Bupropion has been the most prescribed antidepressant by psychiatrists for treating depression not associated with anxiety [34]. This medication is not recommended for athletes with eating disorders because it may increase seizure risk [51]. Athletes treated with bupropion have reported performance improvements [52,53]. For this reason, the WADA has included this drug in the Monitoring Program [43].

CBD also exhibits antidepressant activity, primarily due to its action as an agonist of serotonin and dopamine receptors [54,55].

### 4.2. Pharmacotherapy of Anxiety in Athletes

Pharmacological therapy for anxiety in athletes must balance efficacy and minimal performance impact [56]. Table 3 summarizes the rationale for use and risks associated with the use of anxiolytics in athletes.

Sports psychiatrists, as with depression, prefer prescribing SSRIs due to their fewer side effects [26,34].

Despite benzodiazepines (such as alprazolam, diazepam, and lorazepam) being associated with tolerance and dependence, this drug class remains among the most prescribed worldwide for anxiety treatment, particularly for the rapid efficacy [66]. However, current scientific evidence suggests that benzodiazepines do not have ergogenic (performance-enhancing) effects and may even be ergolytic (performance-impairing) [67]. Studies have not shown improvements in physical performance parameters like shooting accuracy in archers or cycling time with benzodiazepine use. Moreover, given the risks and lack of clear benefits, sports medicine physicians generally recommend benzodiazepines only in specific cases and recommend alternative treatments for anxiety and sleep issues in athletes. These alternatives may include psychological therapies, sleep hygiene strategies, and other pharmacological agents with fewer risks and more evidence for their safety and efficacy in the athletic population [63]. Benzodiazepines can cause side effects such as drowsiness, lethargy, fatigue, cognitive impairment, and impaired motor coordination, which can hinder athletic performance, especially in sports requiring fine motor skills [68]. Muscle weakness is also a potential side effect that could increase the risk of injuries. The potential side effects of anxiolytics, such as sedation and impaired coordination, could indirectly impact performance negatively.

Beta-blockers, used in non-athlete patients, are effective for performance anxiety but may further reduce blood pressure and cardiorespiratory capacity in athletes [48]. Moreover, by acting on tremors, they may improve performance in certain sports and are therefore included in the Prohibited List [32].

CBD reduces perceived anxiety before, during, and after stress-inducing events [69,70]. Studies suggest its efficacy in mitigating sports-related performance anxiety, particularly in high-stakes competitions [71]. Additionally, according to Levin et al. (2012), CBD enhances fear extinction and memory reconsolidation [72], effects that could benefit athletes recovering from traumatic injuries and managing post-traumatic stress disorder (PTSD) symptoms [10,41]. Despite its promising anxiolytic and neuroprotective properties, the use of CBD in elite sports must be approached cautiously. Market-available formulations may contain THC traces above the permitted 0.1% threshold, exposing athletes to unintentional doping violations [36]. Quality assurance and third-party testing are, therefore, essential.

### 4.3. Discussion of Results: Trends in Antidepressants, Anxiolytics, and THC

The Italian data (2011–FH2023) show discontinuous use of antidepressants and anxiolytics among athletes [42], with pronounced peaks in 2021 and the FH2023. These spikes align with delayed mental health help-seeking behaviors following pandemic lockdowns, as athletes confronted accumulated stressors from event cancellations, isolation, and disrupted training routines [73,74]. The years 2020 and 2022, characterized by fewer anti-doping tests due to COVID-19 restrictions [75], record the lowest values, suggesting underestimation during the pandemic. However, the subsequent increase reflects a global trend: as reported by the Organisation for Economic Co-operation and Development (OECD), antidepressant consumption has grown steadily in Europe since 2020 [44], with SSRIs and SNRIs as the main drivers [45]. An explanation for this phenomenon may be attributed to the impact of the COVID-19 pandemic on people’s mental well-being [73]. In a study by Mazza et al., 40% of the sample reported suffering from depression after COVID-19, suggesting a correlation between the infection and mental disorders [74]. Among athletes, however, the increase might also stem from greater mental health awareness, though cultural barriers to screening persist [9].

Regarding anxiolytics, particularly benzodiazepines, Agenzia Italiana del Farmaco (AIFA) [76] reported a 4.3% decrease in national consumption in 2023 compared with the previous year. However, the 2020 increase and post-pandemic levels remain higher than those of 2019. Many studies highlight that benzodiazepines do not exhibit ergogenic effects [77], but the risks of sedation can indirectly compromise performance. Indeed, Confédération Mondiale des Activités Subaquatiques (CMAS) has raised concerns about benzodiazepine use among freedivers due to side effects such as dangerous sedation, reduction in or loss of consciousness, cardiorespiratory depression, coma, and insidious loss of consciousness during performance, even though this practice is not classified as doping [78]. Additionally, Zandonai et al. [79] emphasize the issue of benzodiazepine dependence. Indeed, the management of benzodiazepines in athletes requires a rigorous initial risk/benefit assessment, favoring therapeutic alternatives for prolonged treatments. Continuous monitoring is essential to detecting signs of abuse (e.g., high dosages, dependence, and withdrawal symptoms), ensure prescription adherence, and prevent dangerous interactions (e.g., alcohol or opioids) while integrating psychological evaluations and a multimodal approach.

Concerning cannabis consumption by athletes, reports only include data on THC, as it is classified as a doping substance [32], and not on CBD. According to the 2012 report, the prevalence of cannabis derivatives began to rise in 2004, peaking in 2005 and 2007, followed by another increase in 2012. A decline in THC-positive cases is noted from 2013. This reduction from 2013 onwards aligns with the WADA’s increased threshold (150 ng/mL) implemented in May 2013 [38]. This methodological adjustment likely reduced false positives related to residual THC from out-of-competition use and should be accounted for when interpreting temporal trends. Since 2018, the year in which CBD was excluded from the Prohibited List [37], levels have risen again, despite the reduced number of tests during the COVID-19 pandemic. These trends are corroborated internationally by the WADA in 2022, which also identified THC as the most detected active substance among doping agents [80]. In recent years, increased consumption may stem from decriminalization in many countries and greater social acceptance [81], suggesting broader cultural shifts. Multiple studies found no performance enhancements with intentional marijuana use [81]. CBD, while effective against depression and anxiety, has anti-inflammatory and analgesic properties that remain understudied in sports. However, the risk of THC contamination (>0.1%) in CBD formulations exposes athletes to anti-doping violations [82]. Chronic cannabis use has been rarely associated with myocardial infarction in the absence of atherosclerotic disease [83,84,85]. More common is cannabinoid hyperemesis [86], characterized by abdominal pain, nausea, and intractable vomiting, with risks of dehydration and rhabdomyolysis [87]. Some studies link cannabis use to the onset or worsening of mental disorders [88,89]. A study on exercise and cannabis [90] reports amplified subjective experiences, both positive and negative.

Another key finding is the male-dominated THC consumption pattern, aligning with general population data [91]. Historically, substance use in sports has been disproportionately associated with male athletes, reflecting broader socio-cultural norms where male doping practices were often tacitly tolerated [28,92,93]. Consequently, early anti-doping research and testing protocols primarily focused on male populations. However, emerging evidence indicates substantial substance use among female athletes over time. European drug monitoring reports indicate that approximately one-quarter of individuals entering treatment for substance use disorders are women, with females accounting for 20% of drug-related fatalities [28,92,94]. Gender disparities in substance use involve complex interactions between biological factors (e.g., genetics and neurobiology) and socio-cultural influences (e.g., stigma and expectations) [95]. Notably, women exhibit distinct clinical trajectories compared with men, including accelerated progression from initial use to dependence, higher relapse rates, and greater barriers to treatment seeking. These factors may contribute to the underrepresentation of female athletes in both testing samples (only one-third of total tests) and THC-positive cases in our dataset. Cultural stigma remains particularly pronounced for women, potentially suppressing the self-reporting of mental health symptoms and cannabinoid use [96]. Future research must adopt gender-sensitive methodologies to address these gaps.

### 4.4. Limitations and Future Perspectives

The findings of this study must be interpreted in light of several methodological limitations. First, the analyzed data refer exclusively to athletes subjected to anti-doping testing, potentially excluding undetected cases due to reduced control activities (e.g., during the 2020 lockdowns), risking an underestimation of the actual use of medications and substances. A further critical issue lies in the lack of distinction between pharmacological classes (e.g., SSRIs vs. benzodiazepines) and specific active ingredients, as well as the absence of a gender-stratified analysis, which could clarify dynamics related to disparities in access to care or socio-cultural differences. Third, as registry-based observational research, we lacked granular data on prescription indications, therapeutic adherence, or concurrent psychosocial factors (e.g., stigma perceptions). Regarding cannabis, the absence of CBD data and the exclusive focus on THC prevent an assessment of the impact of CBD legalization on athletes’ habits and its therapeutic use versus contamination risks. Finally, the underrepresentation of female athletes may obscure gender-specific trends.

To address these limitations, future research could adopt multidisciplinary approaches. Transnational analyses might compare Italian data with those from countries with divergent anti-doping policies or cannabis regulations, identifying best practices to balance mental health and athletic compliance. In parallel, longitudinal studies could monitor the impact of CBD regulation by integrating toxicological data (e.g., blood levels of THC/CBD) with self-reports to distinguish between therapeutic use and accidental exposure. Finally, gender-sensitive approaches could explore disparities in access to medications by combining pharmaco-epidemiological data with qualitative investigations into cultural barriers, thereby promoting more inclusive guidelines.

## 5. Conclusions

The use of antidepressants and anxiolytics in sports is a complex issue with potential risks and limited evidence of benefits for athletic performance. Our longitudinal analysis of Italian anti-doping data (2011–FH2023) reveals fluctuating trends in the use of these medications, with notable peaks in 2021 and the FH2023, likely influenced by heightened mental health awareness post-COVID-19. Conversely, the sharp decline in reported usage during 2020 and 2022 underscores the pandemic’s disruptive impact on both testing protocols and mental health reporting. The rising prevalence of THC-positive tests, particularly after regulatory adjustments in 2013 and CBD’s legalization in 2018, reflects broader socio-cultural shifts in substance use, despite its ergolytic risks and lack of evidence of performance enhancement.

Factors such as increased awareness of mental health, stress related to competition and training, and potentially easier access to these medications could contribute to an increase in their use among athletes as well. However, without pharmacological class or gender-specific data from anti-doping reports, these interpretations remain hypothetical. The male-dominated THC consumption pattern aligns with general population trends, though the underrepresentation of female athletes in testing samples (one-third of total tests) limits generalizability. Cultural barriers and stigma may also disproportionately affect women’s mental health reporting and care-seeking behavior, warranting dedicated exploration in future research.

It is also important for athletes to be aware that while benzodiazepines and classical antidepressants are not currently prohibited in sports, their use should be medically justified and carefully managed due to the potential for harm and lack of performance enhancement. Clinicians should prioritize evidence-based, athlete-specific strategies, such as SSRIs for depression or non-pharmacological interventions for anxiety, while rigorously evaluating risks like sedation, dependency, or interactions with anti-doping regulations. CBD emerges as an adjunct for anxiety and recovery, yet contamination risks with THC necessitate stringent quality controls. Future guidelines should address CBD contamination risks and gender disparities in testing.

This study underscores the need for holistic, personalized frameworks that integrate mental health care with athletic performance goals. Future policies should promote multidisciplinary collaboration among sports psychiatrists, anti-doping agencies, and athletic organizations to address stigma, improve diagnostic accuracy, and ensure safe pharmacological practices. Longitudinal studies tracking THC/CBD ratios and gender-stratified analyses are critical to refining guidelines and balancing therapeutic efficacy with regulatory compliance.

## Figures and Tables

**Table 1:** Total number of athletes tested for doping from 2011 to FH2023 and number of athletes using non-doping drugs ^1^/anxiolytics or antidepressants per year.

Year	Athletes Tested for Doping	Non-Doping Drugs Declared	Antidepressants and Anxiolytics
2011	1676	1638	29
2012	1521	1362	21
2013	1390	1498	17
2014	1427	1433	23
2015	860	864	15
2016	806	818	8
2017	1211	1164	12
2018	594	684	7
2019	1245	1379	13
2020	395	375	0
2021	1322	1377	27
2022	364	407	0
FH2023	268	352	9

FH2023: First half of 2023. ^1^ Non-doping drugs are defined as those not on the Prohibited list.

**Table 2:** Doping-test positive athletes in Italy from 2011 to FH2023: number of prohibited substances and THC-positive athletes (total number/male/female) per year.

Year	Positive Athletes	Substance Prohibited	THC-Positive Athletes	THC-Positive Male	THC-Positive Female
2011	52	80	14	-	-
2012	52	66	21	-	-
2013	39	60	10	9	1
2014	58	101	3	2	1
2015	25	41	5	5	0
2016	33	22	4	4	0
2017	58	30	3	3	0
2018	15	13	5	5	0
2019	43	33	9	9	0
2020	13	9	4	4	0
2021	38	47	7	6	1
2022	12	14	4	4	0
FH2023	5	5	1	1	0

FH2023: First half of 2023.

**Table 3:** Rationale for use and risks for anxiolytics use in athletes.

Rationale for Use		Risks and Concerns	
Anxiety Reduction	To manage pre-competition anxiety or general anxiety disorders, which can negatively impact focus, decision making, and muscle tension. Sports where athletes are judged, such as gymnastics or figure skating, may be particularly anxiety-inducing [57,58].	Addiction and Abuse	Benzodiazepines have a known potential for dependence and addiction, even at therapeutic doses. Case studies have reported athletes developing severe benzodiazepine addiction [59].
Sleep Enhancement	To combat insomnia, which can be prevalent in athletes due to intense training schedules and competition stress, aiming for better recovery [60].	Adverse Effects	Besides the performance-related side effects, long-term use can lead to impaired psychomotor and cognitive functioning. Overdose and withdrawal can also have severe complications. Increase in the risk of adverse drug interactions [61].
Muscle Relaxation	Some athletes believe benzodiazepines can aid in muscle relaxation and recovery from intense training [62,63].	Masking Pain and Injuries	By reducing anxiety and potentially masking pain, athletes might not recognize or address injuries appropriately, leading to further harm [64].
Pain Management	Although primarily anxiolytic, they might be used for their muscle-relaxant properties in pain management [61,64].	Lack of Scientific Evidence for Benefits	There is limited scientific evidence supporting the notion that benzodiazepines improve athletic performance or recovery [5,65].

## Data Availability

The data that support the findings of this study are available from the corresponding author, F.M., upon reasonable request. The data are not publicly available due to privacy and ethical restrictions.

## Abbreviations

The following abbreviations are used in this manuscript:

AAPC	Average Annual Percent Change
AIFA	Agenzia Italiana del Farmaco
CBD	cannabidiol
CMAS	Confédération Mondiale des Activités Subaquatiques
COVID-19	Coronavirus Disease 19
DSA	Discipline Sportive Associate
EPS	Enti di Promozione Sportiva
FH2023	first half of 2023
FSN	Federazioni Sportive Nazionali
OECD	Organization for Economic Co-operation and Development
PTSD	post-traumatic stress disorder
SNRIs	serotonin–norepinephrine reuptake inhibitors
SSRIs	selective serotonin reuptake inhibitors
TCAs	tricyclic antidepressants
THC	delta-9-tetrahydrocannabinol
TUE	Therapeutic Use Exemption
WADA	World Anti-Doping Agency
WADC	World Anti-Doping Code
